# Influence of K-line on intraoperative and hidden blood loss in patients with ossification of the posterior longitudinal ligament when undergoing unilateral open-door laminoplasty

**DOI:** 10.1186/s13018-020-02181-9

**Published:** 2021-01-09

**Authors:** Yipeng Li, Jia Li, Feng Wang, Linfeng Wang, Yong Shen

**Affiliations:** 1 Department of Orthopaedic Surgery, Third Hospital of Hebei Medical University, Shijiazhuang, 050051 People’s Republic of China; 2 Key Laboratory of Orthopaedic Biomechanics of Hebei Province, Third Hospital of Hebei Medical University, Shijiazhuang, 050051 People’s Republic of China

**Keywords:** Ossification of the posterior longitudinal ligament, K-line, Intraoperative blood loss, Hidden blood loss, Unilateral open-door laminoplasty

## Abstract

**Background:**

The K-line is a virtual straight line that connects the midpoints of the anteroposterior spinal canal diameter from C2 to C7 on a cervical lateral X-ray film. Patients with cervical ossification of the posterior longitudinal ligament (OPLL), in which the peak of the OPLL exceeds the K-line (K-line [-]), are less likely to experience sufficient decompression after laminoplasty compared with patients for whom the OPLL does not exceed the K-line (K-line [+]). This retrospective study investigated the influence of K-line position relative to the OPLL on intraoperative and hidden blood loss during unilateral open-door laminoplasty for OPLL.

**Methods:**

Data were retrospectively analyzed of 108 patients with OPLL who underwent unilateral open-door laminoplasty between April 2015 and March 2018. Patient cases were categorized as K-line (+) or (-). The evaluated perioperative parameters were haematocrit, haemoglobin, intraoperative and hidden blood loss, surgical time, postoperative drainage, and complications. Radiological parameters included ossification occupancy ratio and C2-7 lordosis.

**Results:**

The K-line (+) and K-line (-) groups were statistically comparable with regard to age, gender, body mass index, OPLL classification, medication history, C2-7 lordosis, postoperative haemoglobin and haematocrit, postoperative drainage, hidden blood loss, and complications. The occupying ratio of the K-line (-) group was significantly greater than that of the K-line (+) group (49.5 ± 15.3% cf. 42.3 ± 10.1%; *P* = 0.006), and the intraoperative blood loss was also significantly higher (286 ± 110.5 mL cf. 205.5 ± 98.3 L, *P* = 0.003). The hidden blood loss of the K-line (-) group was higher than that of the K-line (+), but not significantly (295.5 ± 112.6 mL cf. 265.6 ± 103.8 mL; *P* = 0.072).

**Conclusion:**

Intraoperative and hidden blood loss associated with unilateral open-door laminoplasty can be predicted by the spatial relationship of the K-line and osteophyte. This relationship is a simple and practical index that may help surgeons determine the appropriate surgical strategy for patients with OPLL.

## Background

Heterotopic ossification of the posterior longitudinal ligament (OPLL) of the cervical spine causes a series of clinical symptoms. The prevalence of OPLL in Asian countries is 2 to 4% [1–3]. Conservative treatment is usually ineffective, and surgery is often required. For patients with cervical OPLL (ossification ≥ 3 segments), posterior cervical laminectomy or laminoplasty with internal fixation is often selected [4–6].

On a cervical lateral X-ray film, the K-line is a virtual straight line that connects the midpoints of the anteroposterior spinal canal diameter, from C2 to C7. The K-line and its relationship to the OPLL have been used to prognose clinical outcomes. The condition in which the peak of the OPLL extends beyond the K-line is defined as K-line (-). Patients with cervical OPLL categorized as K-line (-) are less likely to experience sufficient decompression after laminoplasty compared with patients for whom the OPLL does not exceed the K-line, i.e., K-line (+) [7].

Either laminectomy or laminoplasty results in serious damage to the posterior cervical soft tissues and muscles with significant blood loss, especially during decorticalization of the lamina and enlargement of the spinal canal. Such blood loss during surgery can cause hemodynamic instability, surgical complications, and even life-threatening conditions. Clinically, the actual total blood loss is significantly greater than the intraoperative blood loss and postoperative drainage only. Therefore, possible hidden haemorrhage in surgical patients should be noted and calculated according to the total and apparent blood loss [8–12].

To our best knowledge, no study has investigated the position of the K-line relative to the OPLL with regard to perioperative blood loss or its potential influence on surgical strategy. To help determine the optimal strategy for patients with cervical OPLL who require laminoplasty, this retrospective study evaluated the influence of the K-line status, (+) or (-), on perioperative blood loss during unilateral open-door laminoplasty for OPLL.

## Methods

The Ethics Committee of the Third Hospital of Hebei Medical University approved this research. The informed consent of patients was not required because the data were anonymized beforehand. All methods were conducted in accordance with the research principles of the Declaration of Helsinki.

From June 2013 to June 2018, 108 patients with cervical OPLL were admitted to the authors’ hospital and treated with unilateral open-door laminoplasty and internal fixation. All patients met the clinical diagnostic criteria for cervical OPLL and suffered from chronic cervical spinal cord compression, mainly presenting as follows: decreased sensation in the limbs and body, decreased muscle strength of both upper limbs, increased muscle tone of both lower limbs, instability of walking (with a sensation of cotton on the soles of the feet), and dysfunction of the bladder and sphincter. Patients with any of the following were excluded from this study: trauma, tumour, cervical fracture, history of previous cervical spine surgery, coagulation disorder, anticoagulants or antiplatelet medications history, or incomplete follow-up data.

### Surgical technique

All the unilateral open-door laminoplasties were performed by the same experienced spine surgeon (Y.S.). The range of intraoperative decompression was from C3 to C6.

After general anaesthesia by endotracheal intubation, the patient was placed prone. A midline incision was made on the posterior neck skin. The cervical paravertebral muscles were dissociated to expose the lamina and spinous process from C2 to C7. The ligamentum flavum was cut off at the cranial and caudal sides of the surgical segment. The spinous processes were also resected at the surgical segment (C3-C6). The specific opening side was chosen as the side with less oppression from the ossific mass. The trench between the lateral mass and lamina was completely created by cutting off the cortex and cancellous bone, with a high-speed cutting burr and Kerrison punch. On the hinge side, the cortex was removed and an incomplete fracture hinge was created by the high-speed cutting burr. A miniplate of appropriate size was placed at each opening lamina. The drain was removed when the drainage fluid for 24 h was less than 30 mL. All the patients wore a rigid cervical collar after surgery, to reduce the risk of hinge fracture and protect the hinge opening. Because prolonged cervical collar fixation may cause axial neck pain, the time depending was limited and depended on the individual situation.

### Clinical and radiographic measures

The demographic data of each patient included age, gender, and body mass index (BMI).

Also noted were the classification of OPLL (continuous, segmental, mixed, and localized), medication history (hypertension, diabetes mellitus, and cardiovascular disease), haematocrit (Hct), haemoglobin (Hb), and operative time. Intraoperative blood loss was considered the sum of the blood in suction containers and soaked sponges. Postoperative drainage consisted of the volume of blood in the drainage bottle.

The method used by Nadler et al. [13] was applied to estimate the preoperative blood volume (PBV) based on gender, height (m), and weight (kg), as PBV = [k_1_ × height^3^] + [k_2_ × weight] + k_3_, where for men (women), k_1_, k_2_, and k_3_ were 0.3669 (0.3561), 0.03219 (0.03308), and 0.6041 (0.1833), respectively. The method of Gross et al. [14] was used to estimate total blood loss (TBL), as TBL = PBV × (Hct_Preop_ − Hct_Postop_)/average (Hct_Preop_ + Hct_Postop_). The method of Sehat et al. [15] was used to calculate the hidden blood loss (HBL), as HBL = total blood loss − measured blood loss. When transfusion was performed, the method was modified to: HBL = TBL + blood infusion volume − measured blood loss.

**Fig. 1.**
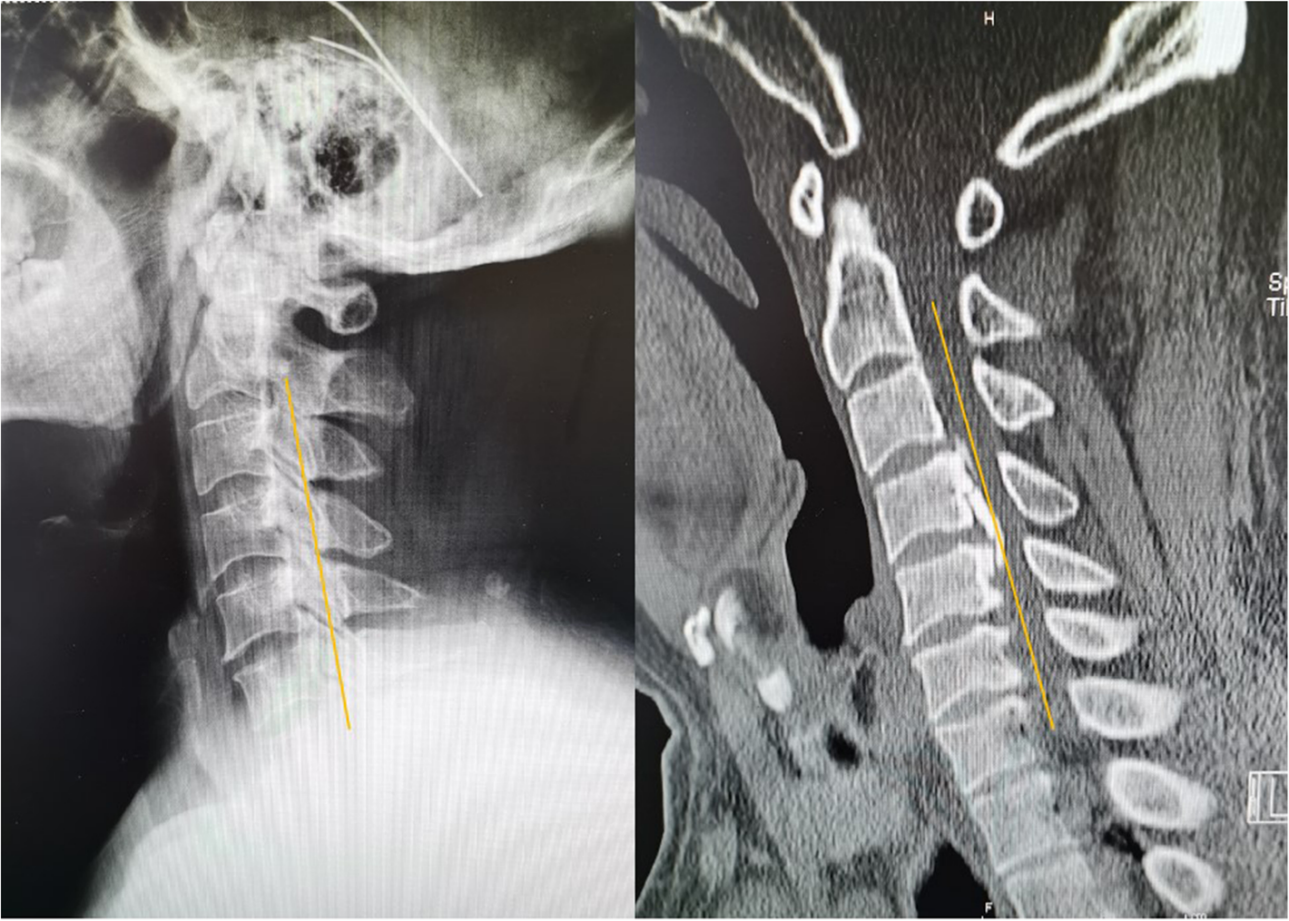
Representative patient in the K-line (-) group. A 60-year-old man. The OPLL mass touched the K-line

**Fig. 2:**
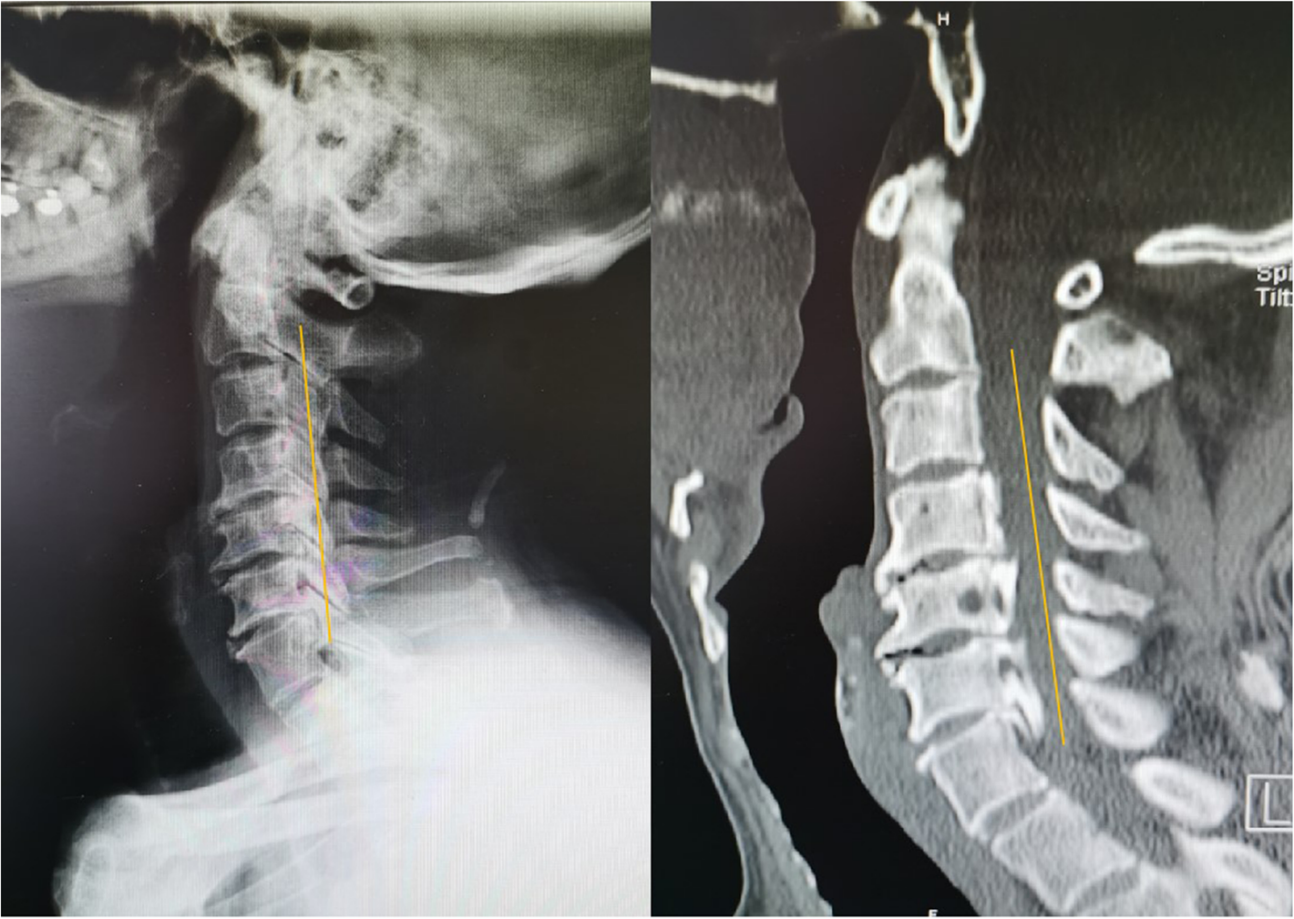
Representative patient in the K-line (+) group. A 49-year-old man. The OPLL mass did not touch the K-line

**Fig. 3:**
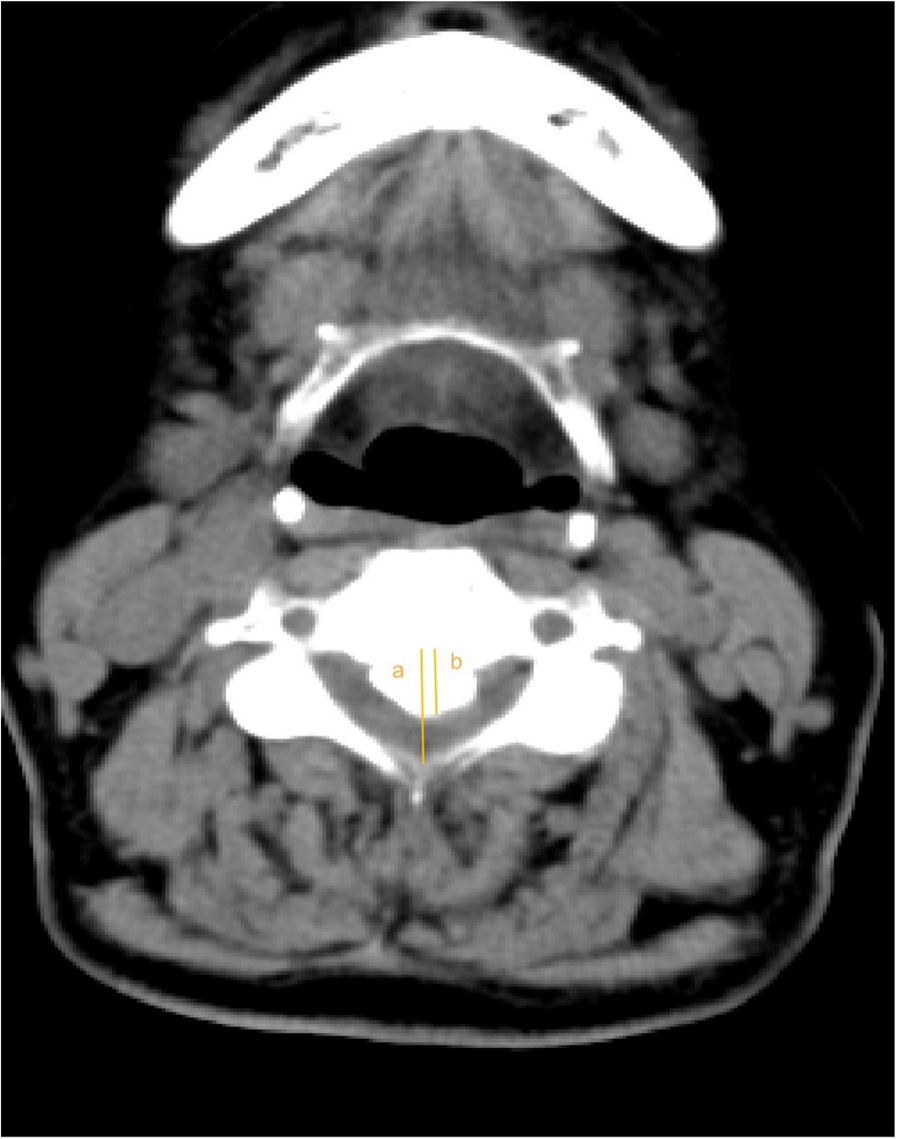
The occupying ratio of the OPLL was calculated as: ossified thickness of the most stenotic area (b)/anteroposterior diameter of the spinal canal (a) × 100%

**Fig. 4:**
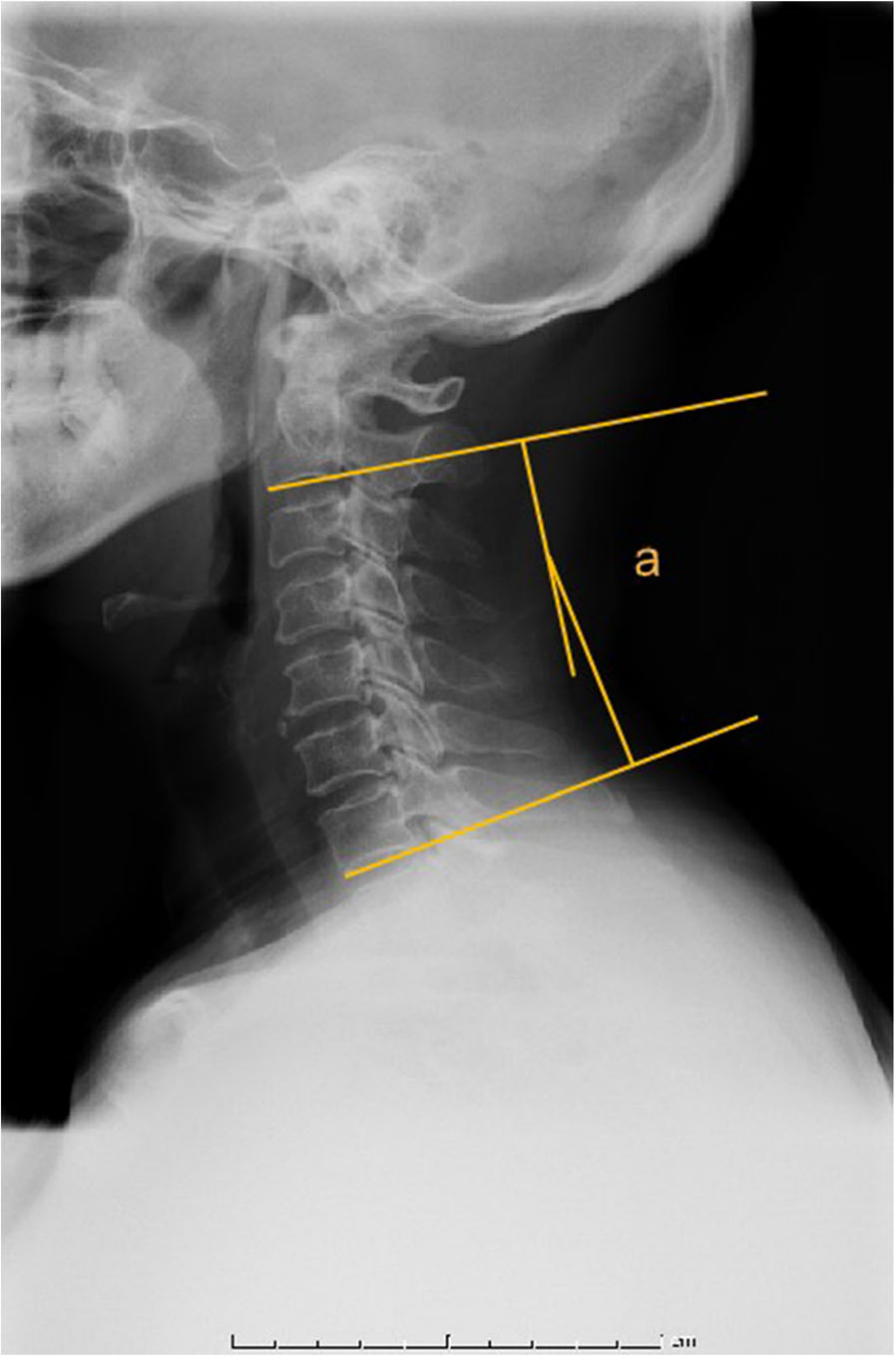
C2-7 lordosis was defined as the angle created by a line parallel to the inferior endplates of the C2 body and a line parallel to that of the C7 body

Radiographic measures included X-ray radiographs, computed tomography, and magnetic resonance imaging. Two independent doctors without knowledge of the clinical outcomes assessed the radiographs. The K-line was defined as a virtual straight line connecting the spinal canal midpoints of C2 and C7 on the cervical lateral radiographs. According to the OPLL and position of the K-line, K-line (+) was defined as a peak of ossification within the K-line, whereas a peak OPLL exceeding the K-line was considered K-line (-), (Figs. 1, 2). The ossification occupancy ratio was defined as the diameter of the OPLL at the thickest ossified part divided by the anteroposterior diameter of the spinal canal (Fig. 3). C2-7 lordosis was identified by the angle created by a line parallel to the inferior endplates of the C2 body and a line parallel to the inferior endplates of the C7 body (Fig. 4).

### Statistical analysis

Statistical analyses were performed using the SPSS statistical software 22.0 for Windows (SPSS, Chicago, IL). All parametric data are presented as mean ± standard deviation. A *P* value < 0.05 was considered statistically significant. Student’s *t* test for continuous variables, or the chi-squared test for dichotomous variables, was used to determine the significance of differences in perioperative parameters between patients in the K-line (+) and K-line (-) groups.

## Results

The data of 108 patients (86 men, 22 women), aged 61.3 ± 10.8 years, were retrospectively reviewed (Table 1). Overall, intraoperative blood loss was 236.8 ± 105.2 mL, and postoperative drainage was 168.7 ± 79.5 mL; hidden blood loss was 276.6 ± 108.1 mL. The surgical time was 101.8 ± 26.9 min.

**Table 1:** Comparison of patient characteristics between K-line (+) group and K-line (-) group

	K-line (+) group	K-line (-)group	*P* value
Age	59.5 ± 11.8	62.8 ± 10.3	0.293
Gender (M/F)	63/12	23/10	0.119
BMI	24.9 ± 3.5	25.4 ± 3.6	0.641
Classification of OPLL			0.748
Continuous	15	7	
Segmental	10	3	
Mixed	39	20	
Localized	11	3	
Medication history			
Hypertension	32 (42.7%)	18 (54.5%)	0.298
Diabetes mellitus	22 (29.3%)	12 (36.4%)	0.505
Cardiovascular disease	15 (20%)	8 (24.2%)	0.618
OOR (%)	42.3 ± 10.1	49.5 ± 15.3	0.006
C2-7 lordosis (°)	12.4 ± 9.7	9.9 ± 10.1	0.217
Preoperative Hb (g/L)	13.2 ± 1.8	13.8 ± 1.6	0.650
Preoperative HCT (%)	40.1 ± 3.2	41.5 ± 2.8	0.598

Regarding the position of the K-line relative to the OPLL, 75 and 33 patients, respectively, were K-line (+) and K-line (-). The ossification occupancy ratio was significantly greater in the K-line (-) group than the K-line (+) group (49.5 ± 15.3% cf. 42.3 ± 10.1%; *P* = 0.006). The K-line (-) and K-line (+) groups were statistically similar with regard to the following: age, gender, BMI, classification of OPLL, medication history, and preoperative Hb and Hct.

No serious complications occurred, such as dural tear, deep vein thrombosis, pulmonary embolism, postoperative hematoma, or cardiovascular accident. Two patients in the K-line (+) group and one patient in the K-line (-) group suffered from incision infection; the wounds healed well after debridement and suture under local anaesthesia. Hypoproteinemia was found in 5 patients in the K-line (+) group and 4 patients in the K-line (-) group.

The intraoperative blood loss in the K-line (-) group was significantly greater than that of the K-line (+) group (286 ± 110.5 mL cf. 205.5 ± 98.3 mL; *P* = 0.003) (Table 2). The postoperative drainage of the 2 groups were comparable (*P* = 0.189): K-line (-), 187.6 ± 88.3 mL; K-line (+) 160.8 ± 70.5 mL. The HBL of the K-line (-) group was slightly higher than that of the K-line (+) group, but the difference was not significant (295.5 ± 112.6 mL cf. 265.6 ± 103.8 mL; *P* = 0.072).

**Table 2:** Comparison of blood loss between K-line (+) group and K-line (-) group

	K-line (+) group	K-line (-) group	*P* value
Intraoperative blood loss (ml)	205.5 ± 98.3	286 ± 110.5	0.003
Postoperative drainage (ml)	160.8 ± 70.5	187:6 ± 88.3	0.189
Hidden blood loss (ml)	265.6 ± 103.8	295.5 ± 112.6	0.072
Postoperative Hb (g/L)	11.1 ± 1.5	10.5 ± 0.8	0.770
Postoperative HCT (%)	32.5 ± 3.8	31.8 ± 3.9	0.598
Surgical time (min)	95.5 ± 20.8	108.3 ± 30.5	0.075

## Discussion

The present study investigated the perioperative blood loss of patients with OPLL undergoing unilateral open-door laminoplasty. Compared with the K-line (+) group, patients in the K-line (-) group experienced significantly higher intraoperative blood loss, but only slightly higher HBL. Thus, patients with OPLL undergoing unilateral open-door laminoplasty with K-line (-) may be more likely to experience major intraoperative blood loss compared with those demonstrating K-line (+).

OPLL often causes spinal stenosis with compression of the spinal cord and nerve roots, resulting in abnormal paraesthesia and motor dysfunction. Posterior surgery is usually chosen for patients with prolonged ossification lesions and severe spinal cord compression, as the risk of anterior surgery is higher and the spinal cord is easily damaged. Fujiyoshi et al. [7] proposed the concept of the K-line to prognose OPLL patients with posterior cervical surgery. The K-line theoretically is a reflection of cervical alignment and the thickness of the ossification foci, two factors that are important in spinal cord compression [7, 16]. Previous studies have reported that open-door laminoplasty could improve the neurological symptoms of patients with OPLL, especially for those in the K-line (+) group relative to those with K-line (-) [17–19].

Yet the comparative differences between K-line (+) and K-line (-) regarding clinical outcomes or blood loss during the open-door laminoplasty have not been sufficiently investigated. Controlling the amount of perioperative blood loss greatly influences patients’ early recovery. Intraoperative blood loss and HBL cannot be ignored during the surgery, as postoperative anaemia will affect the patient’s prognosis, prolong hospitalization, and increase the patient’s economic burden. Therefore, perioperative blood loss must be minimized to better insure postoperative recovery.

Intraoperative blood loss associated with unilateral open-door laminoplasty is mainly due to injury to the internal and external venous plexus and its intervening branches. Previous studies considered that high epidural venous pressure and excessive distribution of epidural veins may be the reason for an increase in intraoperative blood loss. Mathai et al. [20] built a multivariable predictive model of intraoperative blood loss based on 71 patients. They concluded that distribution and adhesion of epidural veins caused by severe spinal stenosis was an important risk factor. Meng et al. [8] analyzed 215 patients who received laminoplasty and suggested that the risk factors for significant intraoperative blood loss and poor recovery of neurologic function were severe spinal stenosis, OPLL, and hinge factures. The only definite risk factor for total blood loss was an occupying ratio ≥ 60%. Kato et al. [21] evaluated intraoperative blood loss in 545 patients who underwent laminoplasty for OPLL and reported that higher occupying ratio was a risk factor for major intraoperative blood loss.

Previous studies have suggested that the specific opening side had no effect on enlargement of the spinal canal, and either side achieved similarly effective decompression of the spinal cord [22, 23]. Choosing the side with less oppression from the OPLL mass reduces spinal cord stimulation and intraoperative blood loss. Our choice of opening side depended upon how best to provide more space for the spinal cord, reduce intraoperative blood loss, and promote backward drift of the spinal cord. In the present study, the intraoperative blood loss in the K-line (-) group was significantly greater than that of the K-line (+) group. This is because the narrower cervical canal of the K-line (-) condition increases the blood pressure of the local veins. Venous return in the cervical canal is reduced, with excessive distribution in the epidural veins. Excessive distribution in the epidural veins caused by an oversized OPLL mass, and adhesion between the vascular structures and ligament, can result in serious blood loss during surgery [24]. When opening the lamina during surgery, intraoperative blood loss may be significant from the fractured side of the lamina and epidural veins. The principal aetiology of ectopic bone formation in OPLL is endochondral ossification, i.e., invading vessels that transport osteoblasts, osteoclasts, and hematopoietic cells to the proliferating cartilage, which leads to formation of the ossification centre. Another factor may be microangiogenesis related to ligament ossification [8, 25, 26].

The specific mechanism of HBL is not clear. Multiple factors during surgery may contribute, such as red blood cell haemolysis, blood entering the interstitial space, and disruption of the fibrinolytic system balance. Other factors include the patient’s gender, age, BMI, number of surgical sections, surgical methods, and operative time [27, 28]. Jiang et al. [12] reported that HBL after laminoplasty could be considerable and threaten patient’s safety, and patients required close attention during the perioperative period. Thick posterior cervical soft tissue was a risk factor for excessive HBL. In the present study, the HBL of the K-line (+) group was slightly lower than that of the K-line (-), but the difference was not significant. However, the sample size may be too small to determine definitively whether the K-line position is a factor affecting HBL.

Strategies for reducing blood loss during spinal surgery have become a focal point of management. Effectively reducing intraoperative blood loss and HBL ensures patients’ hemodynamic stability, reduces allogeneic blood transfusion, clears the surgical vision, and improves the safety and accuracy of the surgery. Good haemostasis also reduces the risk of infection and spinal cord compression caused by postoperative hematoma. A detailed preoperative evaluation should set reasonable strategies for surgical procedures, anaesthesia, and blood transfusion, thereby reducing intraoperative blood loss. A preoperative evaluation mainly considers the risk factors related to perioperative blood loss, coagulation function, and anticoagulation therapy; medication history; and preoperative haemoglobin level. This is especially important for patients with coronary heart, cerebrovascular, or respiratory disease. A history of certain medications can decrease the patient’s tolerance to anaemia. For patients at high risk of much intraoperative blood loss during surgery, haemoglobin levels can be enhanced by preoperative medications such as erythropoietin and iron. It is necessary to monitor clinical signs of preoperative and postoperative blood loss.

If the patient has signs of insufficient organ perfusion or poor oxygenation, but no obvious bleeding, HBL may be suspected; the haemoglobin concentration or red blood cell pressure should be measured. Furthermore, the surgeon’s own experience and skills greatly influence blood loss. All the surgeries in this study were performed by the same group of experienced senior surgeons. Intraoperative blood salvage is recommended. Operators should be mindful of surgical manipulation to reduce bleeding from exposure and prevent complete hinge side fractures when opening the lamina.

## Limitations

There are several limitations in this study. First, it is a retrospective study with a small number of patients in a single hospital, and therefore with a risk of selection bias. Second, the influence of different surgical techniques on total blood loss was not considered. All the patients were prone, and other surgical positions were not included; the effect of surgical position on total blood loss still remains unknown. Furthermore, tranexamic acid reduces perioperative blood loss of spinal surgery, but whether it might reduce the blood loss in patients with K-line (-) is not clear.

## Conclusion

As a simple and practical index, intraoperative blood loss and HBL associated with open-door laminoplasty can be effectively predicted by observing the spatial relationship between the K-line and osteophyte. Therefore, the K-line characteristic is useful for surgeons to determine the appropriate surgical strategy. These findings warrant a prospective study with more patients for confirmation.

## Data Availability

Not applicable.
